# Optimal parameters for final position of teeth in space closure in case of a missing upper lateral incisor

**DOI:** 10.1186/s40510-014-0063-8

**Published:** 2014-11-27

**Authors:** Luca Lombardo, Antonio D′Ercole, Michele Carmelo Latini, Giuseppe Siciliani

**Affiliations:** Postgraduate School of Orthodontics, Ferrara University, Via Montebello, 31, Ferrara, 44121 Italy

**Keywords:** Set up, Missing lateral, Space closure

## Abstract

**Background:**

The aim of this study was to provide clinical indications for the correct management of appliances in space closure treatment of patients with agenesis of the upper lateral incisors.

**Methods:**

Virtual setup for space closure was performed in 30 patients with upper lateral incisor agenesis. Tip, torque and in-out values were measured and compared with those of previous authors.

**Results:**

In the upper dentition, the tip values were comparable to those described by Andrews (Am J Orthod 62(3):296-309, 1972), except for at the first premolars, which require a greater tip, and the first molars, a lesser tip. The torque values showed no differences except for at the canines, where it was greater, and the in-out values were between those reported by Andrews and those by Watanabe et al. (The Shikwa Gakuho 96:209-222, 1996) (except for U3 and U4).

**Conclusions:**

The following prescriptions are advisable: tip 5°, torque 8° and in-out 2.5 for U1; tip 9°, torque 3° and in-out 3.25 for U3; tip 10°, torque −8° and in-out 3.75 for U4; and tip 5°, torque −8° and in-out 4 for U5. Andrews' prescription is suitable for the lower jaw, except for at L6. It is also advisable to execute selective grinding (1.33 ± 0.5 mm) and extrusion (0.68 ± 0.23 mm) on the upper canine during treatment, and the first premolar requires some intrusion (0.56 ± 0.30 mm).

## Background

Agenesis of the upper lateral incisor occurs in roughly 2% of the population [1-4] and comprises 20% of all cases of agenesis [5]. The choice of treatment is influenced by a series of parameters linked to the patient's profile, the type of malocclusion, the shape and size of the teeth and the periodontal biotype [6-9]. There are two major treatment options for upper lateral incisor patients, namely space closure and canine substitution of the missing lateral incisor, or space opening and filling with a prosthetic implant. However, in the anterior sector, the prosthetic option may not be the best solution and cannot be considered a permanent treatment. Indeed, although single implants have relatively long lifespan, they may give rise to biological complications in the long term, for instance, an increase in infraocclusion progression rate [10-14], blue colouring of the labial gingiva [15], abutment exposure [12] and distal papilla recession [15,16]. Hence, space closure is the preferred option for many dentists.

Nevertheless, this solution does present some problems, predominantly in clinical management of the anterior sector. In particular, issues may arise in terms of correct levelling of the marginal gingival contours, as well as achieving the right degree of angulation and inclination on the crowns [8,17-20]. In an ideal upper gingival line, the gingival margins of the central incisors and canines are at the same level [18], with the gingival contours at the lateral incisors being roughly 1 mm lower than the line between these. To prevent an unsightly gingival line, therefore, it is necessary to extrude the canine and, at the same time, intrude the first premolar [19]. In practical terms, this means either that first-, second- and third-order bends will need to be applied to the wire or that the brackets will have to be repositioned several times.

Although in the past many researchers have addressed the various methods of treatment used in lateral incisor agenesis [6-9,21], the literature published to date contains no study using digital setup software to evaluate the optimal parameters for space closure. We have conducted this study with the aim of providing evidence-based clinical indications for good clinical management of these cases. The idea was to exploit digital setup technology to calculate such parameters in patients with lateral incisor agenesis treated by means of space closure, calculating the optimal tip, torque and in-out for each tooth in both arches, as well as the amount of selective grinding to be performed on the palatal surface of the canine after extrusion, the inter-premolar and inter-molar distances of the treated arches - controlling the change in arch shape after setup - and, finally, the amount of canine extrusion and premolar intrusion necessary to create an optimal gingival line.

## Methods

### Sample characteristics

Thirty Caucasian patients with lateral incisor agenesis were selected. The sample comprised 16 males and 14 females of mean age 19.6 (SD 4.8). Initial plaster models and bite wafers were collected for each patient, and selection was performed according to the following criteria: unilateral or bilateral agenesis of the upper lateral incisor, presence of all other teeth except for the second (lost prematurely in some patient) and third molars, absence of other agenetic teeth, complete eruption, absence of bridges or implants, ectopic teeth and supernumerary teeth. In our sample, the mean overjet was 2.11 mm (SD 2.15) and the mean overbite 2.12 mm (SD 2.31).

The plaster models of each patient were scanned using an optical 3D scanner (reVeng Orthodontic, Vision USA Dentrex Company, Cherry Hill, NJ, USA) to obtain three-dimensional virtual models in STL. Numerous studies have confirmed the reliability and precision of today's digital methods of handling virtual models [22-26].

NemoCast 3D software (Nemotec, Madrid, Spain) was used to prepare the models and their setups. The models were oriented in 3D Cartesian space (*x*, *y* and *z*) so that the ideal occlusal plane was in the frontal view, parallel to the *x* and *y* axes and orthogonal to the *z* axis. In the occlusal view, the ideal plane of the median raphe is parallel to the *z* and *y* axes and orthogonal to the *x* axis.

Three landmarks were identified and used to correctly position the occlusal plane:

- The most occlusal tip of the cusp of the most distal tooth on the right
- The most occlusal tip of the cusp of the most distal tooth on the left
- The incisal margin of the central incisors

**Figure 1:**
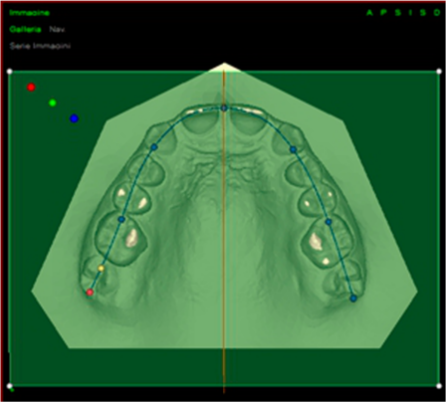
**Determination of the occlusal plane and median raphe (upper jaw).**

**Figure 2:**
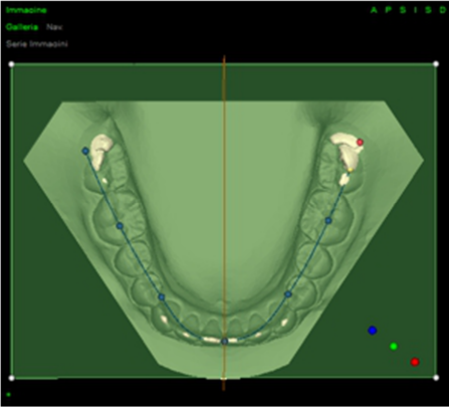
**Determination of the occlusal plane and median raphe (lower jaw).**

Once the occlusal plane was correctly positioned, we identified the median raphe plane perpendicular to the upper occlusal plane, passing through the retro-incisive papilla and the most distal point of the palatine median raphe (Figures 1 and 2).

When creating a setup, the arch form has a key role. We opted for one most similar to that described as ideal by Lombardo and Fattori [27]. Reference points based on the inter-canine and inter-molar distances and the canine and molar depths were used to plot the curve.

A millimetric grid was then positioned on the occlusal plane with the *z* axis coinciding with the palatine median raphe and the point (0;0) which coincides with the incisal margin of the incisors. The arch form curve was then traced through the following five points (Table 1):

**Table 1:** **Arch form**

	**Upper (mm)**	**Lower (mm)**
Inter-canine diameter	39	29
Canine depth	9.1	5.1
Inter-molar diameter	55	51
Molar depth	31.4	26.6

- Point (0;0) on the grid
- Inter-canine diameter point/2 (*x*); right and left canine depths (*y*)
- Inter-molar diameter point/2 (*x*); right and left molar depths (*y*)

The last step in the preparation phase involved segmentation of the models. This was performed by identifying the most mesial and distal points of each tooth in the occlusal view and allowing the software to create the corresponding tooth axis automatically. The crowns were then segmented from the gingiva at the gingival sulcus.

**Figure 3:**
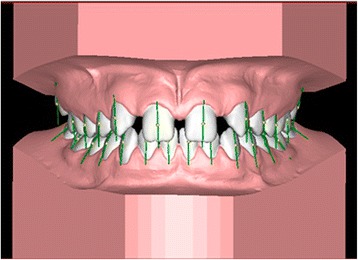
**Placement of FA point and FACC axis.**

Subsequently, as described by Andrews [28], the facial axis (FA) points and their respective facial axes of the clinical crowns (FACC) were identified using both the frontal and occlusal views for each tooth (Figure 3).

**Figure 4:**
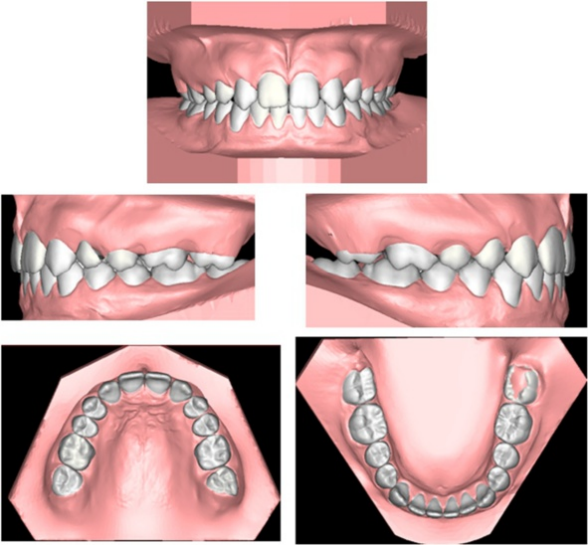
**Final setup (in different views).**

The digital setup was then constructed using the following steps (Figure 4):

- Alignment of the lower arch according to the pre-established arch form
- Flattening of the lower curve of Spee
- Programming the tip and torque values suggested by Andrews for the lower teeth
- Alignment of the upper arch according to the identified arch form and space closure
- Flattening of the upper curve of Spee
- Programming the tip and torque values suggested by Andrews for the upper teeth
- Individual adjustment to create correct overbite and overjet (between 1.5 and 2 mm) of anterior and posterior sectors and correct inter-cuspidation of the molars
- Positioning of the canines and first premolars according to the indications suggested by Zachrisson and Rosa [8,19], i.e. extrusion of the canine and intrusion of the first premolar to create the ideal gingival architecture, and canine torque most similar to that of a lateral incisor to eliminate the canine eminence, which would instead be re-created at the first premolar, increasing the negative torque

**Figure 5:**
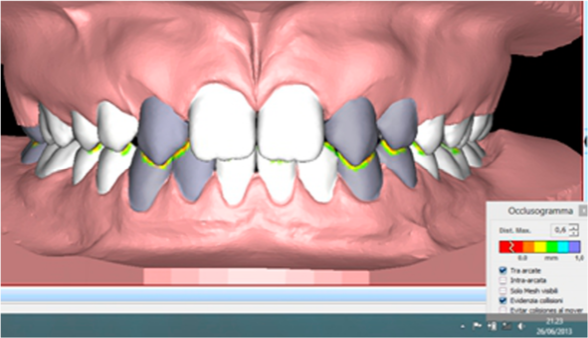
**Occlusal interferences.** The selected teeth are represented in grey.

Finally, by means of the ‘occlusogram’ function (allowing to calculate the amount of superimposition between the upper and lower arches), all intra- and inter-arch collisions were eliminated. The software then provided the tip and torque values for each tooth on the final setup, reading the FACC values with respect to the occlusal plane. The in-out values were then calculated by means of the software's ‘linear measures’ function, using a method similar to that described by Andrews [28]. Each model was sectioned in the occlusal view on the horizontal plane up to the areas of contact between the teeth. On this image, the segments uniting the most vestibular mesial and distal points with respect to the area of contact of each tooth were identified. Another linear measurement was used to connect the FA point with the respective segment identified previously. Once each setup was complete, the ‘occlusogram’ function was used to calculate the amount of grinding in millimetres required at the inevitable pre-contacts between the palatal surface of the upper canines and the lower lateral incisors and canines (Figure 5).

**Figure 6:**
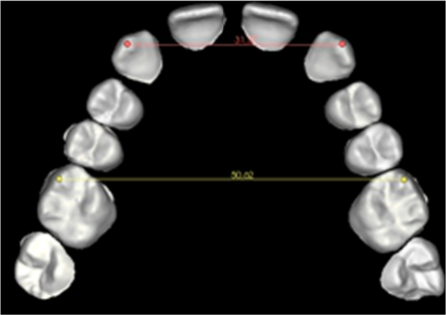
**Measurement of the inter-canine and inter-molar diameters before setup.**

**Figure 7:**
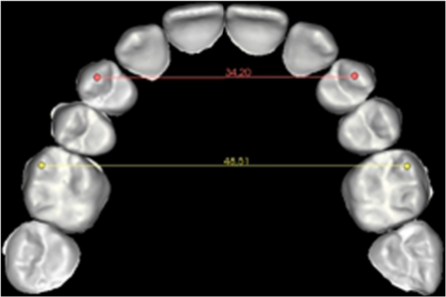
**Measurement of the inter-canine and inter-molar diameters after setup.**

Evaluation of the difference in transversal width at both the canines and molars before and after setup was then performed using the ‘linear measures’ function, calculating the inter-canine and inter-molar diameters of the upper arch. The pre-setup measurements were taken between the cusps of the canines and the mesiovestibular cusps of the molars, respectively. The post-setup inter-canine distance was calculated by measuring the distance between the vestibular cusps of the first premolars and the inter-molar distance between their mesiovestibular cusps (Figures 6 and 7). The ‘linear measures’ function was then used to measure the amount of canine extrusion and first premolar intrusion by analysing the pre- and post-setup FA point positions. The same function was used to calculate the post-setup Bolton index.

### Statistical analysis

Statistical analysis of the following values was performed using the R program [29] and the pwr R package [30]:

- Tip
- Torque
- In-out
- Quantity of selective grinding on upper canine
- Inter-canine and inter-molar diameters
- Amount of canine extrusion and first premolar intrusion
- Post-setup Bolton index

A standard confidence interval was calculated for tip, torque and in-out values using *t*-test statistics. These confidence intervals enabled us to assess the uncertainty of our estimated values. We then used power analysis to compare our tip, torque and in-out values with those of other studies. In essence, power analysis enabled us to assess the minimum effect size that can be detected given an *α* level of confidence of 95% and a *β* power and a specific sample size. Usually, a *β* power parameter of 80% is used as a threshold. As a rule of thumb, *d* effect size of around 0.25 is deemed small, around 0.5 is deemed moderate and over 0.8 is deemed large. To perform the pairwise study comparison required to compare the estimates of pairs of studies, and therefore a power analysis on two independent samples, we used the *t*-test (Table 2). This table includes the column ‘empiricalD’ that reports empirical effect size between the two comparing studies. ‘MinD’ shows the minimum effect size that our sample size could assess within. The last column ‘difference’ assesses whether the differences between the current value and those of other authors can be statistically significant using stated significance and power level.

**Table 2:** **Comparison of the current study tip torque and in-out values with other studies**

**Measure**	**Author**	**EmpiricalD**	**MinD**	**Difference**	**Measure**	**Author**	**EmpiricalD**	**MinD**	**Difference**
U1 Tip	Andrews	0.73	0.58	Y	U1 TQ	Andrews	0.43	0.58	N
U1 Tip	Watanabe et al.	1.16	0.57	Y	U1 TQ	Watanabe et al.	1.61	0.57	Y
U1 Tip	Sebata	0.35	0.68	N	U1 TQ	Sebata	0.45	0.68	N
U1 Tip	Currim and Wadkar	0.64	0.62	Y	U1 TQ	Currim and Wadkar	0.63	0.62	Y
U1 Tip	Doodamani et al.	0.32	0.59	N	U1 TQ	Doodamani et al.	10.79	0.59	Y
U3 Tip	Andrews	0.07	0.58	N	U3 TQ	Andrews	2.72	0.58	Y
U3 Tip	Watanabe et al.	0.49	0.57	N	U3 TQ	Watanabe et al.	2.11	0.57	Y
U3 Tip	Sebata	0.25	0.68	N	U3 TQ	Sebata	0.71	0.68	Y
U3 Tip	Currim and Wadkar	1.47	0.62	Y	U3 TQ	Currim and Wadkar	1.85	0.62	Y
U3 Tip	Doodamani et al.	1.48	0.59	Y	U3 TQ	Doodamani et al.	13.75	0.59	Y
U4 Tip	Andrews	4.28	0.58	Y	U4 TQ	Andrews	0.08	0.58	N
U4 Tip	Watanabe et al.	2.77	0.57	Y	U4 TQ	Watanabe et al.	0.45	0.57	N
U4 Tip	Sebata	1.98	0.68	Y	U4 TQ	Sebata	0.33	0.68	N
U4 Tip	Currim and Wadkar	1.64	0.62	Y	U4 TQ	Currim and Wadkar	0.05	0.62	N
U4 Tip	Doodamani et al.	8.68	0.59	Y	U4 TQ	Doodamani et al.	0.59	0.59	Y
U5 Tip	Andrews	1.20	0.58	Y	U5 TQ	Andrews	0.07	0.58	N
U5 Tip	Watanabe et al.	0.06	0.57	N	U5 TQ	Watanabe et al.	0.33	0.57	N
U5 Tip	Sebata	0.32	0.68	N	U5 TQ	Sebata	0.34	0.68	N
U5 Tip	Currim and Wadkar	0.00	0.62	N	U5 TQ	Currim and Wadkar	0.26	0.62	N
U5 Tip	Doodamani et al.	2.79	0.59	Y	U5 TQ	Doodamani et al.	1.03	0.59	Y
U6 Tip	Andrews	1.28	0.58	Y	U6 TQ	Andrews	0.91	0.58	Y
U6 Tip	Watanabe et al.	0.91	0.57	Y	U6 TQ	Watanabe et al.	1.25	0.57	Y
U6 Tip	Sebata	0.60	0.68	N	U6 TQ	Sebata	2.01	0.68	Y
U6 Tip	Currim and Wadkar	0.48	0.62	N	U6 TQ	Currim and Wadkar	0.61	0.62	N
U6 Tip	Doodamani et al.	1.83	0.59	Y	U6 TQ	Doodamani et al.	0.21	0.59	N
L1 Tip	Andrews	1.25	0.58	Y	L1 TQ	Andrews	0.10	0.58	N
L1 Tip	Watanabe et al.	0.17	0.57	N	L1 TQ	Watanabe et al.	0.48	0.57	N
L1 Tip	Sebata	1.29	0.68	Y	L1 TQ	Sebata	0.94	0.68	Y
L1 Tip	Currim and Wadkar	1.36	0.62	Y	L1 TQ	Currim and Wadkar	0.79	0.62	Y
L1 Tip	Doodamani et al.	1.56	0.59	Y	L1 TQ	Doodamani et al.	8.37	0.59	Y
L2 Tip	Andrews	1.33	0.58	Y	L2 TQ	Andrews	0.38	0.58	N
L2 Tip	Watanabe et al.	0.04	0.57	N	L2 TQ	Watanabe et al.	0.50	0.57	N
L2 Tip	Sebata	1.65	0.68	Y	L2 TQ	Sebata	0.68	0.68	Y
L2 Tip	Currim and Wadkar	1.38	0.62	Y	L2 TQ	Currim and Wadkar	0.76	0.62	Y
L2 Tip	Doodamani et al.	1.87	0.59	Y	L2 TQ	Doodamani et al.	8.60	0.59	Y
L3 Tip	Andrews	0.76	0.58	Y	L3 TQ	Andrews	0.29	0.58	N
L3 Tip	Watanabe et al.	0.25	0.57	N	L3 TQ	Watanabe et al.	0.09	0.57	N
L3 Tip	Sebata	0.76	0.68	Y	L3 TQ	Sebata	1.41	0.68	Y
L3 Tip	Currim and Wadkar	1.65	0.62	Y	L3 TQ	Currim and Wadkar	0.46	0.62	N
L3 Tip	Doodamani et al.	1.43	0.59	Y	L3 TQ	Doodamani et al.	6.95	0.59	Y
L4 Tip	Andrews	1.02	0.58	Y	L4 TQ	Andrews	0.33	0.58	N
L4 Tip	Watanabe et al.	0.38	0.57	N	L4 TQ	Watanabe et al.	0.19	0.57	N
L4 Tip	Sebata	0.16	0.68	N	L4 TQ	Sebata	0.26	0.68	N
L4 Tip	Currim and Wadkar	0.99	0.62	Y	L4 TQ	Currim and Wadkar	0.36	0.62	N
L4 Tip	Doodamani et al.	1.29	0.59	Y	L4 TQ	Doodamani et al.	5.92	0.59	Y
L5 Tip	Andrews	1.52	0.58	Y	L5 TQ	Andrews	0.25	0.58	N
L5 Tip	Watanabe et al.	0.06	0.57	N	L5 TQ	Watanabe et al.	0.13	0.57	N
L5 Tip	Sebata	0.77	0.68	Y	L5 TQ	Sebata	0.04	0.68	N
L5 Tip	Currim and Wadkar	0.77	0.62	Y	L5 TQ	Currim and Wadkar	0.35	0.62	N
L5 Tip	Doodamani et al.	1.61	0.59	Y	L5 TQ	Doodamani et al.	5.42	0.59	Y
L6 Tip	Andrews	1.96	0.58	Y	L6 TQ	Andrews	0.27	0.58	N
L6 Tip	Watanabe et al.	0.84	0.57	Y	L6 TQ	Watanabe et al.	0.23	0.57	N
L6 Tip	Sebata	0.11	0.68	N	L6 TQ	Sebata	1.14	0.68	Y
L6 Tip	Currim and Wadkar	1.09	0.62	Y	L6 TQ	Currim and Wadkar	0.38	0.62	N
L6 Tip	Doodamani et al.	2.22	0.59	Y	L6 TQ	Doodamani et al.	6.76	0.59	Y
In out U1	Andrews	1.19	0.58	Y	In out L1	Andrews	0.36	0.58	N
In out U1	Watanabe et al.	0.58	0.57	Y	In out L1	Watanabe et al.	1.74	0.57	Y
In out U1	Currim and Wadkar	0.30	0.62	N	In out L1	Currim and Wadkar	0.93	0.62	Y
In out U3	Andrews	1.25	0.58	Y	In out L2	Andrews	0.81	0.58	Y
In out U3	Watanabe et al.	1.82	0.57	Y	In out L2	Watanabe et al.	0.34	0.57	N
In out U3	Currim and Wadkar	0.39	0.62	N	In out L2	Currim and Wadkar	0.29	0.62	N
In out U4	Andrews	3.07	0.58	Y	In out L3	Andrews	1.00	0.58	Y
In out U4	Watanabe et al.	2.43	0.57	Y	In out L3	Watanabe et al.	1.58	0.57	Y
In out U4	Currim and Wadkar	0.91	0.62	Y	In out L3	Currim and Wadkar	0.66	0.62	Y
In out U5	Andrews	3.46	0.58	Y	In out L4	Andrews	1.45	0.58	Y
In out U5	Watanabe et al.	1.47	0.57	Y	In out L4	Watanabe et al.	2.15	0.57	Y
In out U5	Currim and Wadkar	1.14	0.62	Y	In out L4	Currim and Wadkar	0.81	0.62	Y
In out U6	Andrews	3.85	0.58	Y	In out L5	Andrews	2.71	0.58	Y
In out U6	Watanabe et al.	0.59	0.57	Y	In out L5	Watanabe et al.	1.54	0.57	Y
In out U6	Currim and Wadkar	1.41	0.62	Y	In out L5	Currim and Wadkar	1.22	0.62	Y
					In out L6	Andrews	3.42	0.58	Y
					In out L6	Watanabe et al.	1.13	0.57	Y
					In out L6	Currim and Wadkar	1.90	0.62	Y

TQ, torque; Y, yes; N, no.

## Results

Tables 3 and 4 show the means and standard deviations of the inclination and angulation values of the crowns following setup. The angulation of the teeth in the upper jaws was positive in all cases. The first premolar displayed the highest degree of tip, followed by the canine. The lowest tip value was measured at the first molar. Torque values in the upper jaw were only positive at the central incisor and the canine, while the other teeth presented progressively diminishing inclination up to the first molar. In the lower jaw, all tip values were positive, with the first molar, followed by the canine, displaying the greatest tip and the central, preceded by the lateral incisor, the smallest. As regards the torque, all values were negative, and a growing trend from the central incisor to the first molar was noted.

**Table 3:** **Mean tip values**

	**Mean (mm)**	**SD (mm)**
Tip U1	4.78	1.11
Tip U3	8.60	2.04
Tip U4	10.20	1.99
Tip U5	5.08	2.88
Tip U6	3.12	2.46
Tip L1	2.13	1.19
Tip L2	2.32	1.35
Tip L3	4.90	2.52
Tip L4	3.18	1.66
Tip L5	4.03	2.44
Tip L6	5.32	2.95

**Table 4:** **Mean torque values**

	**Mean (degree)**	**SD (degree)**
Torque U1	7.68	1.33
Torque U3	3.15	1.19
Torque U4	−8.18	2.31
Torque U5	−8.50	2.10
Torque U6	−15.30	4.94
Torque L1	−1.18	0.79
Torque L2	−1.40	0.81
Torque L3	−11.52	1.07
Torque L4	−17.45	1.33
Torque L5	−22.37	1.71
Torque L6	−32.15	3.41

Table 5 shows the in-out values of each tooth in the upper and lower arches. In both arches, the in-out values tended to increase progressively from the central incisor to the first molar. The mean value of selective canine grinding identified on the setups was 1.33 mm (SD 0.54). Comparison of the pre- and post-setup inter-canine transversal measurements (Table 6) revealed a tendency for the inter-canine diameter to increase and the inter-molar diameter to decrease during the (virtual) treatment. Standard confidence intervals for tip, torque and in-out values are shown in Table 7 (L95 and U95 represent 95% confidence interval).

**Table 5:** **Mean in-out values**

	**Mean (mm)**	**SD (mm)**
In-out U1	2.41	0.42
In-out U3	3.17	0.43
In-out U4	3.73	0.50
In-out U5	3.90	0.56
In-out U6	4.71	0.69
In-out L1	1.49	0.29
In-out L2	1.90	0.31
In-out L3	2.79	0.48
In-out L4	3.39	0.56
In-out L5	3.73	0.63
In-out L6	4.70	0.74

**Table 6:** Inter-canine and inter-molar diameters before and after setup

		**Mean (mm)**	**SD (mm)**
Inter-canine distance	Pre-setup	29.34	3.78
	Post-setup	34.95	1.50
Inter-molar distance	Pre-setup	49.15	2.69
	Post-setup	47.50	1.19

**Table 7:** Standard confidence intervals for tip, torque and in-out values

**Measure**	**L95**	**Mean (°)**	**U95**
U1 Tip	4.37	4.78	5.19
U2 Tip	NA	NA	NA
U3 Tip	7.84	8.60	9.36
U4 Tip	9.46	10.20	10.94
U5 Tip	4.00	5.08	6.16
U6 Tip	2.20	3.12	4.04
L1 Tip	1.69	2.13	2.57
L2 Tip	1.82	2.32	2.82
L3 Tip	3.96	4.90	5.84
L4 Tip	2.56	3.18	3.80
L5 Tip	3.12	4.03	4.94
L6 Tip	4.22	5.32	6.42
U1 TQ	7.18	7.68	8.18
U2 TQ	NA	NA	NA
U3 TQ	2.71	3.15	3.59
U4 TQ	−9.04	−8.18	−7.32
U5 TQ	−9.28	−8.50	−7.72
U6 TQ	−17.14	−15.30	−13.46
L1 TQ	−1.47	−1.18	−0.89
L2 TQ	−1.70	−1.40	−1.10
L3 TQ	−11.92	−11.52	−11.12
L4 TQ	−17.95	−17.45	−16.95
L5 TQ	−23.01	−22.37	−21.73
L6 TQ	−33.42	−32.15	−30.88
In-out U1	2.26	2.42 (mm)	2.58
In-out U2	NA	NA	NA
In-out U3	3.01	3.17	3.33
In-out U4	3.54	3.73	3.92
In-out U5	3.69	3.90	4.11
In-out U6	4.45	4.71	4.97
In-out L1	1.38	1.49	1.60
In-out L2	1.78	1.90	2.02
In-out L3	2.61	2.79	2.97
In-out L4	3.18	3.39	3.60
In-out L5	3.49	3.73	3.97
In-out L6	4.42	4.70	4.98

TQ, torque; NA, not applicable.

Table 2 show the statistical comparison of current study tip, torque and in-out values with other studied values. The mean value of canine extrusion was found to be 0.68 mm (SD 0.23 mm), whereas the mean intrusion of the first premolar was 0.56 mm (SD 0.30 mm). Finally, the mean anterior Bolton index of our sample is reported in Table 8.

**Table 8:** **Anterior and total Bolton index**

	**Mean**	**SD**
Sum mandibular ‘anterior’	33.41 mm	1.99 mm
Sum maxillary ‘anterior’	42.06 mm	2.37 mm
Anterior Bolton index	79.49	3.71
Sum mandibular ‘total’	79.86 mm	4.06 mm
Sum maxillary ‘total’	91.12 mm	5.09 mm
Total Bolton index	87.76	4.15

## Discussion

The first consideration regards the choice of the sample. Dental casts of patients with unilateral or bilateral agenesis of the upper lateral incisors were selected. The selection of casts of patients with full dentition and the removal of the lateral incisors would not have produced setups corresponding to the real-world clinical situation, because the mesio-distal diameters of crowns in patients with both unilateral and bilateral upper lateral agenesis were significantly reduced (except for the upper sixth) [31]. Therefore, since there is no difference in the amount of mesio-distal width reduction between patients with unilateral and bilateral agenesis [31], the prescriptions proposed in this study can be considered valid for both cases.

**Figure 8:**
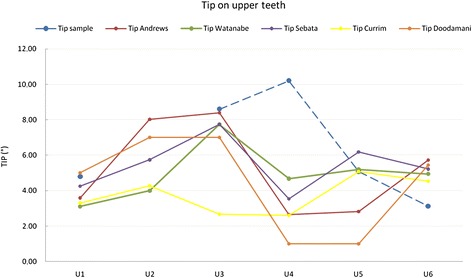
**Comparison with literature values for tip on upper teeth.**

We wanted to reproduce Andrews' method of analysis using NemoCast 3D software, exploiting the greater precision of the digital system. Comparing our values with those obtained by Andrews [28], Watanabe et al. [32], Sebata [33], Currim and Wadkar [34] and Doodamani et al. [35] (Table 2), we can see that the prescription values at U1 and U3 are very similar, especially according with Sebata’s values. However, the U4 tip is far greater than those reported by all the other authors, as the premolar has been used to replace the canine, which usually has a greater tip. The second premolar, U5, had a mean value comparable to those reported by Watanabe et al., Sebata and Currim and Wadkar, but greater than those measured by Andrews and Doodamani et al. Conversely, the tip on U6 was lower than that described by Andrews, Watanabe et al. and Doodamani et al. as, considering class II malocclusion, we had to reduce the tip on this tooth in order to achieve correct inter-cuspidation (Figure 8).

**Figure 9:**
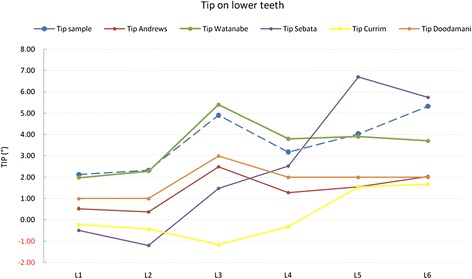
**Comparison with literature values for tip on lower teeth.**

As regards the tip in the lower arch, our mean values were all (except for L6) comparable to those recorded by Watanabe et al., showing the same trend as those measured by Andrews and Doodamani et al. but with slightly higher values (Figure 9), a discrepancy presumably due to our different measurement method. Sebata and Currim and Wadkar showed different trends. However, these differences were not found to be clinically significant, as a difference in prescription of 2° to 3° would be unlikely to create significant clinical implications. The exception to this rule was the L6, which, due to the class II malocclusion, requires a far greater tip than the values reported by the other authors (except for Sebata).

**Figure 10:**
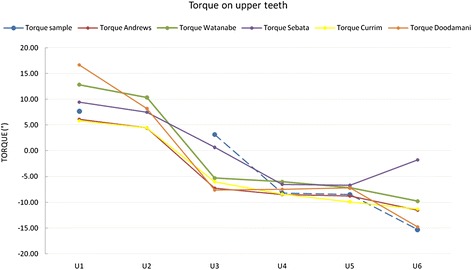
**Comparison with literature values for torque on upper teeth.**

**Figure 11:**
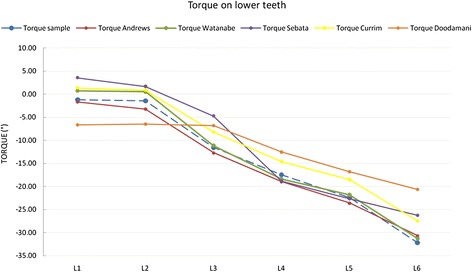
**Comparison with literature values for torque on lower teeth.**

As for torque (Figure 10), our upper arch values were comparable to those of Andrews, Sebata and Currim and Wadkar, except for U3, whose torque was markedly greater in our measurements. However, this is likely to be related to the fact that in the position of the lateral incisor, the canine must have a positive rather than a negative torque. Our lower arch values were all comparable to those reported in the literature (Figure 11).

**Figure 12:**
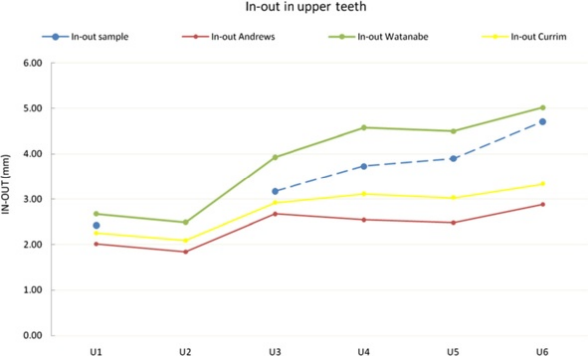
**Comparison with literature values for in-out on upper teeth.**

**Figure 13:**
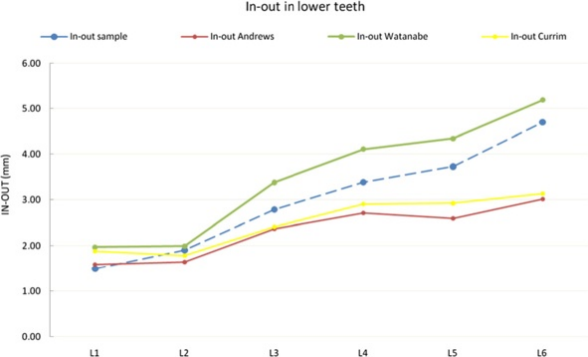
**Comparison with literature values for in-out on lower teeth.**

Our in-out values in the upper arch fell between those reported by Andrews and those by Watanabe et al. (Figure 12). The canine in place of the lateral incisor displays higher values with respect to the missing tooth due to its greater labiolingual dimensions, but the difference observed between our U1 and U3 was comparable to that described by Andrews. In contrast, U4 in-out was greater than that reported by Andrews and Currim and Wadkar, as it was used to create the canine eminence. Hence, the difference in in-out between U3 and U4 (Δ U3 − U4) was greater than that calculated by Andrews. The main differences found are summarized in Table 9. In terms of lower arch in-out, once again, our values fell between those of Andrews and Watanabe et al., displaying a comparable trend (Figure 13).

**Table 9:** **In-out values (upper arch)**

**In-out values**				**Delta (mm)**
Andrews' in-out values				
U1	2.01	U3	2.67	0.66
U3	2.67	U4	2.54	- 0.13
U4	2.54	U5	2.48	- 0.06
Current study in-out values				
U1	2.41	U3	3.17	0.76
U3	3.17	U4	3.73	0.56
U4	3.73	U5	3.90	0.17

The other objective of our study was to determine the amount of selective grinding to be performed on the palatal surface of the upper canine. This investigation yielded clear results that were very similar on the left and right, namely that 1.33 mm of selective grinding is sufficient to prevent pre-contacts with the lower arch during the course of treatment.

Analysis of the inter-arch diameters showed that the mean final U3 inter-canine diameter was comparable with that reported by Lombardo et al. [36], while the U6 diameter was statistically smaller. This discrepancy is undoubtedly ascribable to the mesialization of the posterior sector, meaning that, overall, the inter-arch diameters are as expected.

Regarding canine extrusion and first premolar intrusion, to achieve an ideal gumline and prevent gingival displacement of the canine gingival zenith [17], the canine must be extruded by 0.68 mm and the first premolar intruded by 0.56 mm.

We also calculated the total and anterior Bolton indices [37]. We analysed both monolateral and bilateral agenesis, finding that the mesio-distal widths of teeth in agenesis patients are reduced (except for U6). Hence, the mean total ratio in our sample was 87.76 (SD 4.15), less than that proposed by Bolton (91.3, SD 1.91) and indicating the presence of a maxillary excess. This discrepancy could be caused by the fact that while calculating the maxillary sum, we replaced U2, which has a mesio-distal diameter of 6.5 mm, with U7, whose mesio-distal diameter is 9 mm [38]. Furthermore, according to our findings, the U6 is the only tooth whose mesio-distal diameter is not reduced in agenesis patients, which could also help explain the total maxillary excess we found. In contrast, we measured a mean anterior Bolton ratio of 79.49 (SD 3.71), which compared to the Bolton index (77.2, SD 1.65) lets us assume an upper anterior deficiency. In bilateral agenesis cases, the anterior maxillary sum is generally calculated using U4 instead of the missing U2, whose mesio-distal widths are almost equal in normal conditions [38] (7 mm U4, 6.5 mm U2). However, in cases of monolateral agenesis (48% of our sample), the contralateral incisor is often microdontic [31] (mean width of our sample U2 is 4.54 mm, SD 0.79) considerably reducing the maxillary sum, which is likely to explain the mean anterior maxillary defect.

## Conclusions

According to our findings, the following positioning prescriptions are indicated in the upper arch (for both unilateral and bilateral agenesis):

- Central incisor: tip 5°, torque 8° and in-out 2.5 mm.
- Canine: tip 9°, torque 3° and in-out 3.25 mm (0.75 mm greater with respect to U1).
- First premolar: tip 10°, torque −8° and in-out 3.75 mm (0.50 mm greater with respect to U3).
- Second premolar: tip 5°, torque −8° and in-out 4 mm (0.25 mm greater with respect to U4).
- First molar: a tube with −15° of torque can be used.
- In the lower arch, Andrews' prescriptions can be used on all teeth except for the first molar, which instead requires a tip of 5°.

During treatment, it is advisable to selectively grind the palatal surface of the upper canine by 1.33 ± 0.5 mm in order to prevent pre-contacts, which may slow treatment down. We also suggest extruding the canine by 0.68 ± 0.23 mm and intruding the first premolar by 0.56 ± 0.30 mm in order to obtain ideal gingival architecture.
